# 8,15-Dioxa-10,13-di­aza­tetra­cyclo­[14.4.0.0^2,7^.0^9,14^]icosa-1(16),2,4,6,9(14),10,12,17,19-nona­ene

**DOI:** 10.1107/S1600536813011318

**Published:** 2013-04-30

**Authors:** Thothadri Srinivasan, Venkatesan Kalpana, Perumal Rajakumar, Devadasan Velmurugan

**Affiliations:** a Centre of Advanced Study in Crystallography and Biophysics, University of Madras, Guindy Campus, Chennai 600 025, India; b Department of Organic Chemistry, University of Madras, Guindy Campus, Chennai 600 025, India

## Abstract

The asymmetric unit of the title compound, C_16_H_10_N_2_O_2_, contains one half-mol­ecule, the complete mol­ecule being generated by twofold rotation symmetry. The plane of the pyrazine ring forms a dihedral angle of 64.87 (6)° with that of the benzene ring, and the planes of the two benzene rings are inclined to one another by 54.20 (6)°. The O atom deviates from the plane of the benzene ring by 0.1549 (8) Å. There are no significant inter­molecular inter­actions in the crystal.

## Related literature

For applications of the pyrazine ring system in drug development, see: Du *et al.* (2009 ▶); Dubinina *et al.* (2006 ▶); Ellsworth *et al.* (2007 ▶); Mukaiyama *et al.* (2007 ▶). For background to the fluorescence properties of related compounds, see: Kawai *et al.* (2001 ▶); Abdullah (2005 ▶) and for their biological activity, see: Seitz *et al.* (2002 ▶); Temple *et al.* (1970 ▶). For a related structure, see: Nasir *et al.* (2010 ▶).
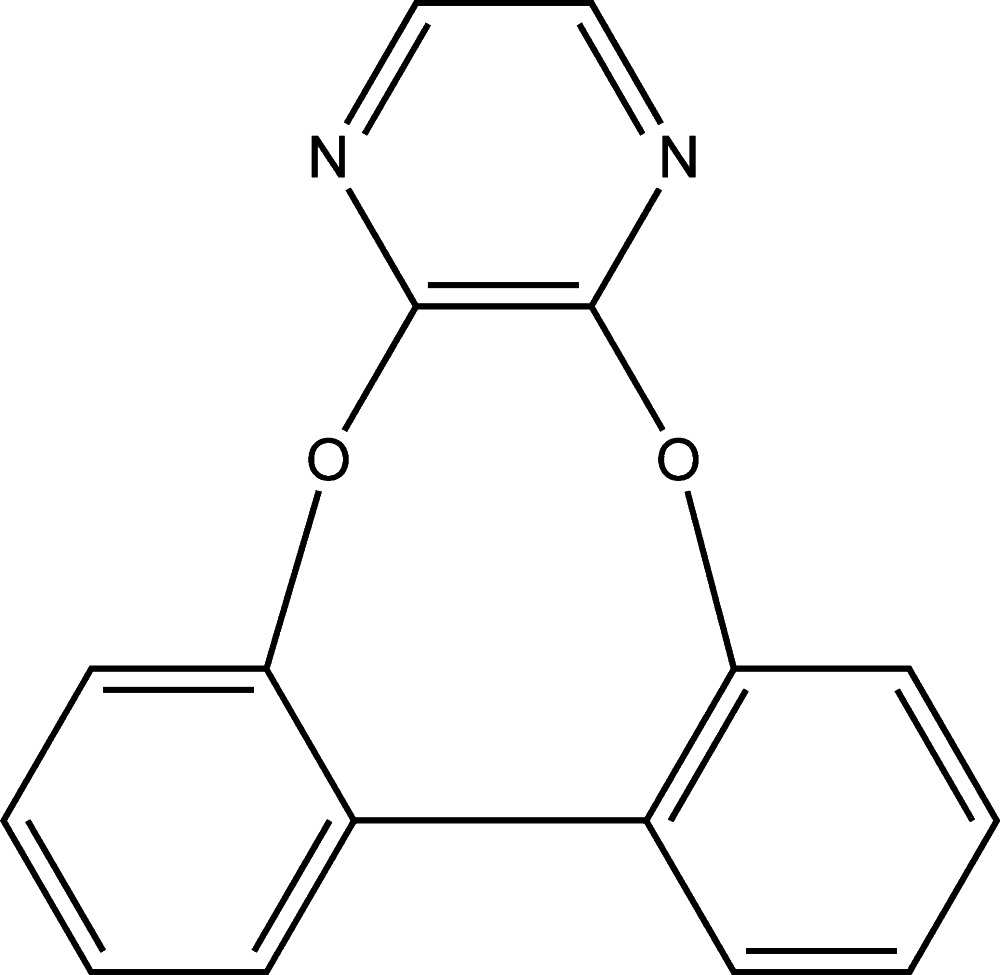


## Experimental

### 

#### Crystal data

C_16_H_10_N_2_O_2_
*M* *_r_* = 262.26
Orthorhombic, 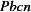

*a* = 14.429 (3) Å
*b* = 10.162 (2) Å
*c* = 8.3313 (18) Å
*V* = 1221.6 (4) Å^3^
*Z* = 4
Mo *K*α radiation
μ = 0.10 mm^−1^
*T* = 293 K
0.30 × 0.25 × 0.20 mm

#### Data collection

Bruker SMART APEXII area-detector diffractometer
Absorption correction: multi-scan (*SADABS*; Bruker, 2008 ▶) *T*_min_ = 0.972, *T*_max_ = 0.981
6082 measured reflections
1502 independent reflections
1226 reflections with *I* > 2σ(*I*)
*R*_int_ = 0.029

#### Refinement

*R*[*F* ^2^ > 2σ(*F* ^2^)] = 0.033
*wR*(*F* ^2^) = 0.106
*S* = 1.03
1502 reflections
92 parameters
H-atom parameters constrained
Δρ_max_ = 0.22 e Å^−3^
Δρ_min_ = −0.13 e Å^−3^

### 

Data collection: *APEX2* (Bruker, 2008 ▶); cell refinement: *SAINT* (Bruker, 2008 ▶); data reduction: *SAINT*; program(s) used to solve structure: *SHELXS97* (Sheldrick, 2008 ▶); program(s) used to refine structure: *SHELXL97* (Sheldrick, 2008 ▶); molecular graphics: *ORTEP-3 for Windows* (Farrugia, 2012 ▶) and *Mercury* (Macrae *et al.*, 2008 ▶); software used to prepare material for publication: *SHELXL97* and *PLATON* (Spek, 2009 ▶).

## Supplementary Material

Crystal structure: contains datablock(s) global, I. DOI: 10.1107/S1600536813011318/su2586sup1.cif

Structure factors: contains datablock(s) I. DOI: 10.1107/S1600536813011318/su2586Isup2.hkl

Supplementary material file. DOI: 10.1107/S1600536813011318/su2586Isup3.cml

Additional supplementary materials:  crystallographic information; 3D view; checkCIF report

## Figures and Tables

**Fig. 1.**
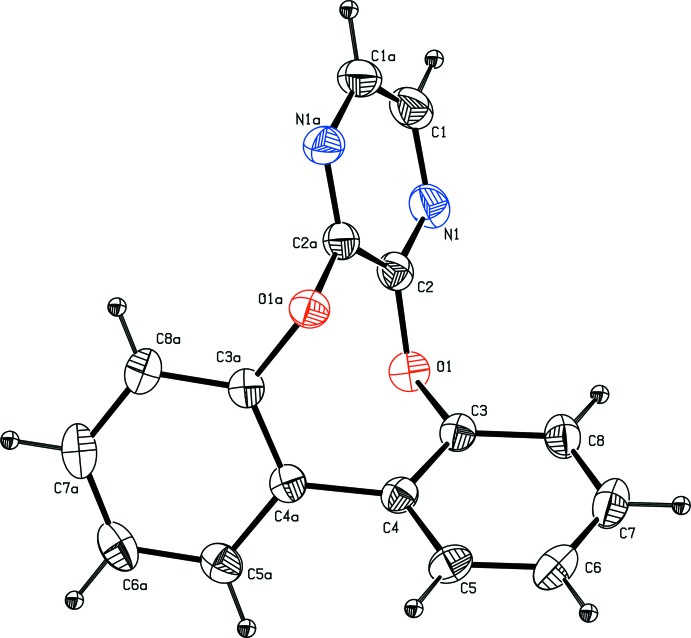
The molecular structure of the title molecule, with atom labelling (symmetry code: (a) -x+1, y, -z+1/2). Displacement ellipsoids are drawn at the 30% probability level.

## supplementary crystallographic information

### Comment

The pyrazine ring system is a useful structural element in medicinal chemistry and has found broad applications in drug development which can be used as antiproliferative agent (Dubinina *et al.*, 2006), potent CXCR3 antagonists (Du *et al.*, 2009),CB1 antagonists (Ellsworth *et al.*, 2007) and c-Src inhibitory (Mukaiyama *et al.*, 2007). On-going structural studies of heterocyclic N-containing derivatives (Nasir *et al.*, 2010) are motivated by an investigation of their fluorescence properties (Kawai *et al.*, 2001; Abdullah, 2005). Pyrazine derivatives were shown to display antimycobacterial (Seitz *et al.*, 2002) and potential antimalarial (Temple *et al.*, 1970) activities. In view of different applications of this class of compounds, we have undertaken the crystal structure determination of the title compound.

The title compound, Fig. 1, contains one half molecule in the asymmetric unit; the complete molecule is generated by a two-fold rotation axis, about [010]; symmetry code:(i) -x+1, y, -z+1/2.

The central pyrazine ring (C1/N1/C2/C1^i^/N1^i^/C2^i^) forms a dihedral angle of 64.87 (6) ° with the phenyl ring (C3-C8). The dihedral angle between the symmetry related phenyl rings (C3-C8) and (C3^i^-C8^i^) is 54.20 (6) °. The deviation of the atom O1 from the phenyl ring (C3-C8) is 0.1549 (8) Å.

In the crystal, there are no significant intermolecular interactions present.

### Experimental

To a stirred solution of Cs_2_CO_3_(15 mmol) in CH_3_CN (5 mL), was added dropwise independently a solution of corresponding diol (10 mmol) in CH_3_CN (25 mL) and a solution of 2,3-dichloropyrazine (10 mmol) in CH_3_CN (25 mL). The reaction mixture was stirred at reflux for 12 h. The reaction mixture was allowed to cool to room temperature and then poured into water (200 mL), and extracted with CH_2_Cl_2_ (2X100 mL). The combined organic layers were washed with water (100 mL), brine (50 mL) and dried over Na_2_SO_4_. The solvent was evaporated and the crude product was purified by column chromatography with CHCl_3_ as an eluent to give the title compound. Single crystals suitable for X-ray diffraction were obtained by slow evaporation of a solution of the title compound in hexane at room temperature.

### Refinement

The hydrogen atoms were placed in calculated positions and refined in the riding model approximation: C—H = 0.93 Å with *U*_iso_(H) = 1.2*U*_eq_(C).

### Crystal data

**Table:**

C_16_H_10_N_2_O_2_	*F*(000) = 544
*M**_r_* = 262.26	*D*_x_ = 1.426 Mg m^−^^3^
Orthorhombic, *P**b**c**n*	Mo *K*α radiation, λ = 0.71073 Å
Hall symbol: -P 2n 2ab	Cell parameters from 1502 reflections
*a* = 14.429 (3) Å	θ = 2.5–28.3°
*b* = 10.162 (2) Å	µ = 0.10 mm^−^^1^
*c* = 8.3313 (18) Å	*T* = 293 K
*V* = 1221.6 (4) Å^3^	Block, colourless
*Z* = 4	0.30 × 0.25 × 0.20 mm

### Data collection

**Table:**

Bruker SMART APEXII area-detector diffractometer	1502 independent reflections
Radiation source: fine-focus sealed tube	1226 reflections with *I* > 2σ(*I*)
Graphite monochromator	*R*_int_ = 0.029
ω and φ scans	θ_max_ = 28.3°, θ_min_ = 2.5°
Absorption correction: multi-scan (*SADABS*; Bruker, 2008)	*h* = −18→19
*T*_min_ = 0.972, *T*_max_ = 0.981	*k* = −5→13
6082 measured reflections	*l* = −7→10

### Refinement

**Table:**

Refinement on *F*^2^	Secondary atom site location: difference Fourier map
Least-squares matrix: full	Hydrogen site location: inferred from neighbouring sites
*R*[*F*^2^ > 2σ(*F*^2^)] = 0.033	H-atom parameters constrained
*wR*(*F*^2^) = 0.106	*w* = 1/[σ^2^(*F*_o_^2^) + (0.0544*P*)^2^ + 0.1721*P*] where *P* = (*F*_o_^2^ + 2*F*_c_^2^)/3
*S* = 1.03	(Δ/σ)_max_ < 0.001
1502 reflections	Δρ_max_ = 0.22 e Å^−^^3^
92 parameters	Δρ_min_ = −0.13 e Å^−^^3^
0 restraints	Extinction correction: *SHELXL97* (Sheldrick, 2008), Fc^*^=kFc[1+0.001xFc^2^λ^3^/sin(2θ)]^-1/4^
Primary atom site location: structure-invariant direct methods	Extinction coefficient: 0.012 (3)

### Special details

**Table:**

Geometry. All esds (except the esd in the dihedral angle between two l.s. planes) are estimated using the full covariance matrix. The cell esds are taken into account individually in the estimation of esds in distances, angles and torsion angles; correlations between esds in cell parameters are only used when they are defined by crystal symmetry. An approximate (isotropic) treatment of cell esds is used for estimating esds involving l.s. planes.
Refinement. Refinement of F^2^ against ALL reflections. The weighted R-factor wR and goodness of fit S are based on F^2^, conventional R-factors R are based on F, with F set to zero for negative F^2^. The threshold expression of F^2^ > 2sigma(F^2^) is used only for calculating R-factors(gt) etc. and is not relevant to the choice of reflections for refinement. R-factors based on F^2^ are statistically about twice as large as those based on F, and R- factors based on ALL data will be even larger.

### Fractional atomic coordinates and isotropic or equivalent isotropic displacement parameters (Å^2^)

**Table:**

	*x*	*y*	*z*	*U*_iso_*/*U*_eq_	
C1	0.53624 (10)	1.14890 (12)	0.19682 (18)	0.0623 (4)	
H1	0.5600	1.2289	0.1616	0.075*	
C2	0.53746 (8)	0.92738 (11)	0.19677 (13)	0.0426 (3)	
C3	0.60493 (7)	0.71770 (11)	0.24296 (13)	0.0395 (3)	
C4	0.54878 (7)	0.61036 (10)	0.27797 (12)	0.0390 (3)	
C5	0.58736 (8)	0.50933 (12)	0.36919 (14)	0.0480 (3)	
H5	0.5523	0.4349	0.3922	0.058*	
C6	0.67748 (9)	0.51846 (14)	0.42609 (16)	0.0568 (4)	
H6	0.7020	0.4509	0.4883	0.068*	
C7	0.73073 (8)	0.62674 (15)	0.39094 (15)	0.0594 (4)	
H7	0.7908	0.6328	0.4305	0.071*	
C8	0.69529 (8)	0.72654 (12)	0.29713 (15)	0.0505 (3)	
H8	0.7317	0.7989	0.2706	0.061*	
N1	0.57395 (7)	1.03683 (10)	0.14200 (14)	0.0546 (3)	
O1	0.57275 (5)	0.81298 (8)	0.13462 (9)	0.0446 (2)	

### Atomic displacement parameters (Å^2^)

**Table:**

	*U*^11^	*U*^22^	*U*^33^	*U*^12^	*U*^13^	*U*^23^
C1	0.0751 (9)	0.0430 (6)	0.0688 (9)	−0.0077 (6)	−0.0246 (6)	0.0065 (6)
C2	0.0464 (6)	0.0431 (6)	0.0383 (6)	−0.0018 (4)	−0.0088 (4)	0.0017 (4)
C3	0.0383 (5)	0.0458 (5)	0.0344 (5)	0.0021 (4)	0.0021 (4)	−0.0019 (4)
C4	0.0382 (5)	0.0429 (5)	0.0357 (5)	0.0033 (4)	0.0052 (4)	−0.0026 (4)
C5	0.0498 (6)	0.0476 (6)	0.0467 (6)	0.0092 (5)	0.0103 (5)	0.0029 (5)
C6	0.0547 (7)	0.0697 (8)	0.0461 (6)	0.0241 (6)	0.0024 (5)	0.0049 (6)
C7	0.0417 (6)	0.0870 (10)	0.0496 (7)	0.0130 (6)	−0.0066 (5)	−0.0073 (7)
C8	0.0394 (6)	0.0623 (7)	0.0497 (7)	−0.0040 (5)	−0.0005 (5)	−0.0078 (6)
N1	0.0593 (6)	0.0495 (6)	0.0548 (6)	−0.0094 (5)	−0.0118 (5)	0.0090 (5)
O1	0.0486 (5)	0.0467 (4)	0.0384 (4)	−0.0009 (3)	0.0040 (3)	0.0041 (3)

### Geometric parameters (Å, º)

**Table:**

C1—N1	1.3423 (17)	C4—C5	1.3935 (16)
C1—C1^i^	1.371 (3)	C4—C4^i^	1.483 (2)
C1—H1	0.9300	C5—C6	1.3871 (17)
C2—N1	1.3124 (15)	C5—H5	0.9300
C2—O1	1.3707 (14)	C6—C7	1.374 (2)
C2—C2^i^	1.398 (2)	C6—H6	0.9300
C3—C8	1.3826 (15)	C7—C8	1.3787 (18)
C3—C4	1.3897 (15)	C7—H7	0.9300
C3—O1	1.4029 (13)	C8—H8	0.9300
			
N1—C1—C1^i^	121.95 (8)	C6—C5—H5	119.6
N1—C1—H1	119.0	C4—C5—H5	119.6
C1^i^—C1—H1	119.0	C7—C6—C5	120.33 (12)
N1—C2—O1	116.01 (10)	C7—C6—H6	119.8
N1—C2—C2^i^	122.05 (7)	C5—C6—H6	119.8
O1—C2—C2^i^	121.79 (6)	C6—C7—C8	120.18 (11)
C8—C3—C4	122.16 (11)	C6—C7—H7	119.9
C8—C3—O1	118.51 (10)	C8—C7—H7	119.9
C4—C3—O1	118.93 (9)	C7—C8—C3	119.14 (11)
C3—C4—C5	117.38 (10)	C7—C8—H8	120.4
C3—C4—C4^i^	119.18 (8)	C3—C8—H8	120.4
C5—C4—C4^i^	123.41 (8)	C2—N1—C1	115.99 (12)
C6—C5—C4	120.77 (12)	C2—O1—C3	117.73 (8)
			
C8—C3—C4—C5	−0.82 (16)	C4—C3—C8—C7	−0.97 (17)
O1—C3—C4—C5	171.84 (9)	O1—C3—C8—C7	−173.66 (10)
C8—C3—C4—C4^i^	177.25 (11)	O1—C2—N1—C1	176.77 (10)
O1—C3—C4—C4^i^	−10.09 (16)	C2^i^—C2—N1—C1	1.3 (2)
C3—C4—C5—C6	1.84 (16)	C1^i^—C1—N1—C2	−0.2 (2)
C4^i^—C4—C5—C6	−176.15 (12)	N1—C2—O1—C3	126.38 (10)
C4—C5—C6—C7	−1.07 (18)	C2^i^—C2—O1—C3	−58.13 (16)
C5—C6—C7—C8	−0.78 (19)	C8—C3—O1—C2	−86.29 (12)
C6—C7—C8—C3	1.78 (18)	C4—C3—O1—C2	100.77 (11)

Symmetry code: (i) −*x*+1, *y*, −*z*+1/2.
